# Supplementary material for: Is Xenopus laevis introduction linked with Ranavirus incursion, persistence and spread in Chile?
Source: PeerJ. 2023 Feb 27;11:e14497. doi: 10.7717/peerj.14497 (PMC9979829; doi:10.7717/peerj.14497)
Supplement: Table S1 — Ranavirus presence was tested through real time PCR. Results are presented by host species and study sites. [file peerj-11-14497-s001.docx]

| **Region** | **Site** | **Lat** | **Long** | **Species** | **n** | **Rv +** | **Observed Prevalence** |
| --- | --- | --- | --- | --- | --- | --- | --- |
| Central | Calama | -22.501987 | -68.960164 | *Gambusia holbrooki* | 70 | 0 | 0 |
| Andean dry puna |  |  |  | *Oncorhynchus mykiss* | 11 | 0 | 0 |
| Chilean matorral | Rio Elqui | -29.897250 | -71.244583 | *Gambusia holbrooki* | 82 | 0 | 0 |
| Chilean matorral | Jardin Botanico | -32.039694 | -71.498111 | *Gambusia holbrooki* | 40 | 0 | 0 |
|  | Villa Alemana | -33.036251 | -71.370742 | *Gambusia holbrooki* | 5 | 0 | 0 |
| Chilean matorral | Rancagua | -34.185358 | -70.799575 | *Gambusia holbrooki* | 64 | 0 | 0 |
| Valdivian | Nahuelbuta | -37.882251 | -73.371236 | *Cyprinus carpio* | 3 | 0 | 0 |
| temperate |  |  |  | *Cheirodon galusdae* | 31 | 0 | 0 |
| forests |  |  |  | *Galaxia maculatus* | 70 | 0 | 0 |
| Valdivian  temperate forests | Puyehue | -40.723249 | -72.433281 | *Oncorhynchus mykiss* | 60 | 0 | 0 |
| Valdivian temperate forests | Valdivia | -39.872720 | -73.160637 | *Gambusia holbrooki* | 60 | 0 | 0 |
|  |  |  |  | Total | 496 | 0 | 0 |
