# Supplementary material for: Is Xenopus laevis introduction linked with Ranavirus incursion, persistence and spread in Chile?
Source: PeerJ. 2023 Feb 27;11:e14497. doi: 10.7717/peerj.14497 (PMC9979829; doi:10.7717/peerj.14497)
Supplement: Table S2 — Ranavirus presence was detected through real time PCR. Results are presented by host species and study sites. * Vulnerable. ** Endangered. *** Critically endangered. [file peerj-11-14497-s002.docx]

| **Eco-region** | **Site** | **Lat** | **Long** | **Species** | **n** | **Rv +** | **Observed Prevalence** |
| --- | --- | --- | --- | --- | --- | --- | --- |
| Central | Rio Loa | -22.748641 | -68.071030 | *^***^Telmatobius dankoi* | 50 | 0 | 0 |
| Andean | Calama | -22.748641 | -68.071030 | *Rhinella spinulosa* | 18 | 0 | 0 |
| Puna |  |  |  | *^***^Telmatobius vilamensis* | 10 | 0 | 0 |
|  | Valle de Jere | -23.187166 | -67.991194 | *Rhinella spinulosa* | 30 | 0 | 0 |
| Chilean | Rio Elqui | -29.897250 | -71.244583 | *Pleurodema thaul* | 51 | 0 | 0 |
| matorral |  |  |  | *^*^Calyptocephalella gayi* | 5 | 0 | 0 |
| Valdivian | Nahuelbuta | -37.882251 | -73.371236 | *Batrachyla taeniata* | 4 | 0 | 0 |
| temperate |  |  |  | *^*^Calyptocephalella gayi* | 11 | 0 | 0 |
| forests |  |  |  | *^**^Rhinoderma darwinii* | 8 | 0 | 0 |
|  |  |  |  | *^*^Eusophus contulmoensis* | 11 | 0 | 0 |
|  |  |  |  | *Pleurodema thaul* | 14 | 0 | 0 |
| Valdivian | Puyehue | -40.723249 | -72.433281 | *^**^Rhinoderma darwinii* | 7 | 0 | 0 |
| temperate |  |  |  | *Eusophus calcaratus* | 23 | 0 | 0 |
| forests |  |  |  | *^*^Eusophus vertebralis* | 2 | 0 | 0 |
|  |  |  |  | *Eusophus emiliopigini* | 1 | 0 | 0 |
|  |  |  |  | Total | 245 | 0 | 0 |
