# Supplementary material for: Is Xenopus laevis introduction linked with Ranavirus incursion, persistence and spread in Chile?
Source: PeerJ. 2023 Feb 27;11:e14497. doi: 10.7717/peerj.14497 (PMC9979829; doi:10.7717/peerj.14497)
Supplement: File S1 [file peerj-11-14497-s003.docx]

1. Quality assessment

fastqc filename.fastq

1. Filtering and trimming sequences:

prinseq-lite-0.20.4/prinseq-lite.pl -fastq RV75_Alex_R1_001.fastq -fastq2 RV75_Alex_R2_001.fastq -trim_left 30 -custom_params "CAAGCAGAAGACGGCATACGAGCTCTTCCGATCT";"AC 25" -trim_right 60 -min_qual_score 28 -min_qual_mean 30 -min_len 50 -derep 1 -out_format 3

1. Assembly of filtered and trimmed sequences:

spades.py --phred-offset 33 --only-assembler -1 /Users/castrolab03/Documents/ranas_cvaldivia/bt_results/RV_all_R1_001_prinseq_good_C4Ij.fastq -2 /Users/castrolab03/Documents/ranas_cvaldivia/bt_results/RV_all_R2_001_prinseq_good_t7fe.fastq -o /Users/castrolab03/Documents/ranas_cvaldivia/assemblies

1. Blast of obtained contig against custom database containing Ranavirus genomes:

blastn -query final_contigs.fasta -db ranavirus -outfmt "6 qseqid qlen sseqid slen qstart qend sstart send qseq sseq lenght nident pident evalue" -out blast_rv_genomes_trimmed_contig_k127
