# Supplementary material for: Is Xenopus laevis introduction linked with Ranavirus incursion, persistence and spread in Chile?
Source: PeerJ. 2023 Feb 27;11:e14497. doi: 10.7717/peerj.14497 (PMC9979829; doi:10.7717/peerj.14497)
Supplement: File S2 [file peerj-11-14497-s004.docx]

GACTTGGCCACTTATGACAATCTTGAGAGAGCAATGTACGGGGGTTCGGACGCCACCACGTACTTTGTCAAGGAGCACTACCCCGTGGGGTGGTTCACCAAGCTGCCGTCTCTGGCTGCCAAGATGTCGGGTAACCCGGCTTTCGGGCAGCAGTTTTCGGTCGGCGTTCCCAGGTCGGGGGATTACATCCTCAACGCCTGGTTGGTGCTCAAGACCCCCGAGGTCGAGCTCCTGGCTGCAAACCAGCTGGGAGACAATGGCACCATCAGGTGGACAAAGAACCCCATGCACAACATTGTGGAGAGCGTCACCCTCTCATTCAACGACATCAGCGCCCAGTCCTTTAACACGGCATACCTGGACGCCTGGAGCGAGTACACCATGCCAGAGGCCAAGCGCACAGGCTACTATAACATGATAGGCAACACCAGCGATCTCATCAACCCCGCCCCGGCCACAGGCCAGGACGGAGCCAGGGTCCTCCCGGCCAAGAACCTGGTTCTTCCCCTCCCATTCTTCTTCTCCAGAGAC
